# Predicted optimum ambient temperatures for broiler chickens to dissipate metabolic heat do not affect performance or improve breast muscle quality

**DOI:** 10.1080/00071668.2015.1124067

**Published:** 2016-02-29

**Authors:** I. Zahoor, M.A. Mitchell, S. Hall, P.M. Beard, R.M. Gous, D.J. De Koning, P.M. Hocking

**Affiliations:** ^a^ Roslin Institute and R(D)SVS, University of Edinburgh, Easter Bush, Midlothian, EH25 9RG, UK; ^b^ University of Veterinary and Animal Sciences, Lahore, Pakistan; ^c^ SRUC, Easter Bush, Midlothian, EH25 9RG, UK; ^d^ University of KwaZulu-Natal, Private Bag X01, Scottsville, 3209, South Africa; ^e^ Department of Animal Breeding and Genetics, Swedish University of Agricultural Sciences, 750 07Uppsala, Sweden

## Abstract

1. An experiment was conducted to test the hypothesis that muscle damage in fast-growing broiler chickens is associated with an ambient temperature that does not permit the birds to lose metabolic heat resulting in physiological heat stress and a reduction in meat quality.
2. The experiment was performed in 4 climate chambers and was repeated in 2 trials using a total of 200 male broiler chickens. Two treatments compared the recommended temperature profile and a cool regimen. The cool regimen was defined by a theoretical model that determined the environmental temperature that would enable heat generated by the bird to be lost to the environment.
3. There were no differences in growth rate or feed intake between the two treatments. Breast muscles from birds on the recommended temperature regimen were lighter, less red and more yellow than those from the cool temperature regimen. There were no differences in moisture loss or shear strength but stiffness was greater in breast muscle from birds housed in the cool compared to the recommended regimen.
4. Histopathological changes in the breast muscle were similar in both treatments and were characterised by mild to severe myofibre degeneration and necrosis with regeneration, fibrosis and adipocyte infiltration. There was no difference in plasma creatine kinase activity, a measure of muscle cell damage, between the two treatments consistent with the absence of differences in muscle pathology.
5. It was concluded that breast muscle damage in fast-growing broiler chickens was not the result of an inability to lose metabolic heat at recommended ambient temperatures. The results suggest that muscle cell damage and breast meat quality concerns in modern broiler chickens are related to genetic selection for muscle yields and that genetic selection to address breast muscle integrity in a balanced breeding programme is imperative.

## INTRODUCTION

Modern, rapidly growing strains of meat poultry exhibit an elevated incidence of spontaneous or idiopathic myopathy and an increased susceptibility to stress induced myopathy (Mitchell, 1999; Sandercock *et al*., 2006). These pathologies are attributable ultimately to alterations in intracellular cation regulation and calcium homeostasis (Sandercock, and Mitchell, 2003; Sandercock *et al*., 2009; Sporer *et al*., 2012) and consequent changes in sarcolemmal integrity. A common feature of all myopathic and dystrophic conditions is the leakage of the intracellular muscle enzyme creatine kinase (CK) into the circulation and increased plasma CK activity is a useful diagnostic indicator of muscle pathology and altered sarcolemmal integrity (Mitchell, 1999).

Broiler muscle myopathies are characterised by histological changes indicative of muscle degeneration including hyaline (hypercontracted) fibres, fatty infiltration, fragmentation of the sarcoplasm, mononucleocyte infiltration and focal necrosis. Indicators of tissue regeneration such as basophilic fibres and internalised nuclei have also been observed (Mahon, 1999; MacRae *et al*., 2006, 2007). Pathological changes may be observed in both glycolytic and more oxidative muscles in broilers (MacRae *et al*., 2006) but myopathy is more prevalent in breast (*Pectoralis major*) compared to leg muscles (*Biceps femoris*).

Successful genetic selection for muscle fibre hypertrophy accounts for the majority of increased muscle size in meat birds and many studies have shown a negative correlation between muscle fibre diameter and meat quality (Tůmová, and Teimouri, 2009). Rapidly growing lines of birds also exhibit a reduced thermoregulatory capacity compared to layers and may thus be more susceptible to heat stress and to consequent problems, including muscle damage, acid–base disturbances and reduced meat quality (Mitchell *et al*., 2005). Sandercock *et al*. (2006) showed that broilers were more affected by heat stress than layers at the same age or body weight and that muscle damage was increased by heat stress.

There have recently been reports that a proportion of commercial broiler carcasses exhibit white striping, characterised by white parallel striations in the direction of the muscle fibres (Petracci *et al*., 2013*a*), or “wooden breast muscle” (Sihvo *et al*., 2014). These conditions lead to the condemnation of severely affected carcasses. Pathological studies have shown that muscle abnormalities in samples affected by white striping (Kuttappan *et al*., 2013*b*) are similar to those previously observed in birds under conditions of heat stress (Mitchell, 1999).

Model calculations of the optimum ambient temperature to avoid heat stress in fast-growing broilers and turkeys suggest that birds at currently recommended environmental temperatures will be unable to dissipate enough heat to maximise performance and will therefore be subjected to physiological heat stress (Emmans, 1989; Gous, and Morris, 2005). Consistent with this hypothesis, a recent microarray experiment compared the gene expression profile of heat-stressed layers and broilers with layers and broilers kept at ambient temperature. The gene expression profile of heat stressed layers was similar to that of broilers kept at ambient temperature; however, heat-stressed broilers exhibited a further increase in the expression of similar genes (Zahoor, 2012).

The main objective of this experiment was to test the hypothesis that broilers experience a thermal load that may impose a degree of heat stress under normal thermal conditions and husbandry practices. It is proposed that this may contribute to the development of muscle damage that could be alleviated by rearing them at lower, optimum, ambient temperatures, leading to improved meat quality. The alternative hypothesis would claim that genetic selection has resulted in broilers that are more susceptible to muscle pathology independent of environmental temperature.

## MATERIALS AND METHODS

### Animals and husbandry

A total of 200 1-d-old Ross 308 male broiler chicks were purchased from a commercial hatchery in two batches of 100 birds. The chicks were housed in 4 pens (1 × 2 m, width × depth) in each of 4 climate chambers at 6 or 7 chicks/pen (100 chicks per trial). In Trial 1, 8 or 9 chicks/pen were initially housed in 3 of the climate chambers because the 4th chamber had malfunctioned: Chicks were randomly assigned from the other chambers to the pens in this room at 7 d of age after it had been repaired. The chambers were refurbished and Trial 2 was conducted in exactly the same way except that all 4 pens were used from the start. The pens were littered with wood shavings and feed and water were provided *ad libitum* at all times. The relative humidity was maintained at a steady 60% throughout both trials. Light intensity was 40 lx from 1 to 7 d and 20 lx thereafter. The photoperiod was 23L:1D for the first 24 h and decreased by one hour per day to 18L:6D at 7 d which was maintained to the end of the experiments. A commercial broiler starter feed (12.7 MJ ME/kg; 230 g crude protein (CP)/kg) was fed from 0 to 28 d and a broiler finisher (13.2 MJ ME/kg; 195 CP/kg) from 29 to 42 d.

### Treatments

The climate chambers were maintained at the same temperature to 3 weeks of age (Table 1). Thereafter, two chambers selected at random were kept at the conventional recommended temperatures (Table 1, column 2) and two at a temperature regimen predicted by the EFG broiler growth model (EFG, 2009). The objective of this treatment was to ensure that the heat generated by a male Ross 308 broiler growing at its potential could be successfully lost to the environment (Table 1, column 3). In Trial 2, each chamber was allocated to the alternative temperature treatment.

**Table 1.** Control and low temperature (cool) environmental temperature profiles to which male broiler chickens were subjected at different ages. The control temperature profile was that recommended in the breeder’s manual (Aviagen, 2007). The temperatures of the cool treatment were based on a theoretical model of ambient temperatures required for broilers to exhibit potential protein growth (EFG, 2009). Relative humidity was 70% to day 5 and 60% thereafter in both treatments.

Age (d)	Control (°C)	Cool (°C)
1	30	30
3	28	28
6	27	27
9	26	26
12	25	25
15	24	24
18	23	23
21	22	20.5
24	21	19
27	21	17
30	21	16
33	21	15
36	21	14
39	21	14

### Observations

Weekly pen mean feed intake and body weight were recorded. Three birds from each pen were selected at random to determine individual traits. Rectal temperature was recorded at 41 d (Trial 1) or 39 d (Trial 2) using a thermistor probe (Model 612-849; RS Components Ltd., Corby, Northants, UK) inserted 5 cm into the rectum and maintained in position until the digital readout displayed a constant value. A 1.5 ml blood sample was obtained from a wing vein into a heparinised sample tube, centrifuged within 20 min of collection at 1300 *g* for 4 min at 4°C, the plasma decanted into a fresh tube and stored on dry ice. The samples were subsequently transferred to a laboratory refrigerator and kept at −20°C for later determination of CK activity.

Another three birds were killed at 42 or 44 d (one chamber from each treatment on both days) by an intravenous injection of 2 ml of Euthatal (2 mg sodium pentobarbital/ml, Merial, Harlow, UK). The skin over the left breast muscle was removed and a 1 × 1 × 2 cm sample running parallel to the muscle fibres was removed from the rostral area of the breast and placed in 10% buffered normal formalin. After 15 min the initial pH (pH_i_) of the muscle was recorded at three locations (rostral, medial and caudal) using a Testo 205 pH/Temperature Measuring Instrument (Testo AG, Lenzkirch, Germany) calibrated at pH 7.01 and 4.01. The pH meter was rinsed in deionised water after each reading and cleaned in alcohol after each chamber. The CIE system colour profile (CIE, 1978) was used to determine initial breast colour lightness (L*_i_), redness (a*_i_) and yellowness (b*_i_) at the same three areas using a Chroma Meter CR-410 (Konica Minolta Sensing Inc., Osaka, Japan). The carcasses were placed in plastic bags and stored at −4°C. Each carcass was removed after 24 h and ultimate pH (ph_u_) and colour of the right breast muscle (L*_u_, a*_u_, b*_u_) was determined as described above. A 2 × 1 × 1 cm sample of breast muscle was dissected as described above and stored in a sealed plastic bag, placed on ice and stored at 4°C for subsequent determination of shear strength. A 1 × 1 × 1 cm sample of muscle was similarly obtained for the later measurement of water holding capacity.

Shear strength was measured once on each sample using a materials force transducer (Model LRX, Lloyd Instruments, Bognor Regis, UK) fitted with a Warner–Bratzler shear blade. The muscle samples were sectioned transversely at a speed of 60 mm/min at a maximum load of 400 N to determine the maximum force to fracture and sample stiffness.

The water holding capacity of the breast muscle was determined by placing the weighed sample between two Whatman No.1 filter papers (diameter 12.5 cm) folded in half and set between two discs with a diameter of 47 mm positioned above and below the sample in the LRX materials testing instrument. The upper disc was lowered on to the sample at a rate of 100 mm/min and a maximum load of 400 N was applied to the sample for 15 s. The sample was weighed again and the extruded water loss was calculated as [((pre-weight – post-weight)/pre-weight) × 10] (Petracci *et al*., 2012).

CK activity (mU/ml) was determined using a Biovision CK Activity colorimetric assay kit (K777-100, Milpitas, CA, USA) adapted for use on chicken plasma following the procedure outlined in Mitchell and Sandercock (1995). Briefly, a 1:500 dilution in assay buffer was used and all samples were incubated and measured at 25°C in a spectrophotometer (Multiskan, Thermo Scientific, USA) with kinetic readings, every minute for 40 min at 450 nm. All plate quality controls had recoveries between 90% and 110%, with inter-assay CV% of 17%.

### Histology

A total of 32 breast muscle samples were selected at random (one per pen) for histological examination. Muscle sections were stained with Haematoxylin and Eosin (H&E) by standard procedures and three sections of muscle (two cross sections, one longitudinal section) were mounted on glass slides. Each slide was examined blindly and assessed by one researcher (P.B.). For each slide, an overall score (0–3) was awarded, representing the severity of pathology present in the tissues on the slide: 0, not present; 1, mild change; 2, moderate change; 3, marked change with decimal fractions also being used. The presence of multifocal accumulations of perivascular mononuclear inflammatory cells (lymphocytes and macrophages forming a cuff around a blood vessel), thick cords of perimysial adipose tissue and an estimate of the proportion of acute vs chronic pathological changes present were noted and coded as binary (0,1) data.

### Statistical analyses

The statistical model included block effects for chamber nested within experiment and temperature regimen as the treatment effect. All analyses were conducted using Genstat procedures (Genstat, 2009), for mean pen body weight, feed intake, rectal temperature, CK, muscle quality traits and pathology severity score by analysis of variance. The CK determinations were transformed to natural logarithms to normalise the residual variance. For statistical evaluation of the pathology scores, the presence of adipose tissue cords and mononuclear accumulation were analysed as binary traits with Poisson errors in a Generalised Linear Mixed Model framework, whereas the overall number of birds in different classes of acute and chronic degeneration were analysed as a contingency table by a *χ* ^2^-statistic.

## RESULTS

Mean body weight at 42 d for cool and conventional treatments, respectively, were 3.58 and 3.61 kg (SED, standard error of a difference, 0.97, *P = *0.75); average feed intakes at 35–42 d were 254 and 262 g/d (SED 13.3, *P = *0.64) and feed conversion rates (FCR) were 1.71 and 1.76 (SED 0.141, *P = *0.75). Differences between the treatments for body weight, feed intake and FCR were not significant at any other age from 7 to 35 d (Supplementary Table; online version only). Mortality (back transformed) from 14 to 42 d was 1.21 (23%) and 1.42 (19%) (SED 0.258, *P = *0.54) and occurred largely from 28 to 42 d of age.

Treatment means and tests of significance for the muscle, plasma CK and meat quality traits are given in Table 2. Rectal temperature was lower in the birds exposed to the cool temperature regimen. There was no difference in CK, initial or ultimate pH of the breast muscle. Initial breast muscle was significantly lighter, less red and more yellow in the broilers kept on the conventional compared to the low temperature treatment (*P < *0.05). After 24 h these differences remained although the treatment means for breast muscle yellowness were not significantly different, possibly because individual values were more variable. The means for moisture loss and tenderness (maximum load to shear) were similar whereas breast muscle stiffness was greater in samples from birds in the cool environment (*P < *0.01).

**Table 2.** Mean values and SED of muscle and meat quality traits in male broilers exposed to conventional or cool environmental temperatures from 3 to 6 weeks of age.

Trait	Conventional	Cool	SED	Significance (*P*)
Rectal temperature (°C)	41.4	41.2	0.05	0.025
Creatine kinase, ln mU/ml^a^	5.68 (293)	5.82 (338)	0.112	0.200
pH_i_	6.82	6.84	0.062	0.551
ph_u_	5.92	5.95	0.034	0.530
L*_i_	59.1	57.3	0.40	0.006
a*_i_	12.8	13.8	0.36	0.038
b*_i_	7.18	6.26	0.292	0.025
L*_u_	62.5	60.2	0.87	0.043
a*_u_	11.7	12. 8	0.24	0.007
b*_u_	7.48	6.79	0.446	0.183
Moisture loss (%)	24.5	23.8	0.99	0.556
Maximum load (N)	3.00	2.96	0.044	0.472
Stiffness (N/mm)	5.71	6.92	0.201	0.002

^a^Back-transformed means in parenthesis.

### Muscle pathology

**Figure 1.**
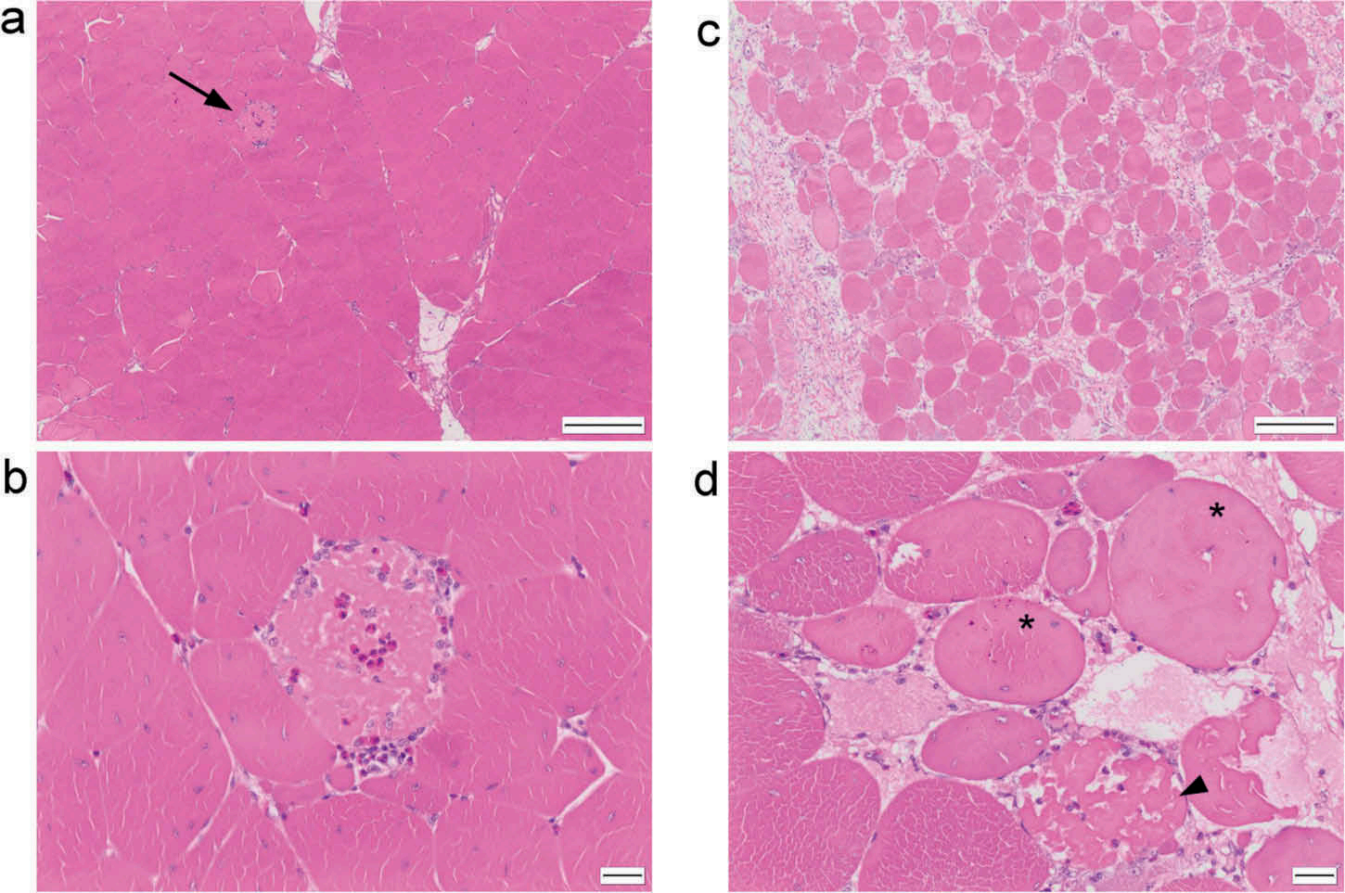
(a) H&E stained section of breast muscle with mild (grade 1) pathology, showing individual myofibre degeneration (arrow). (b) Higher magnification showing fragmented, pale eosinophilic sarcoplasm with infiltration by heterophils and mononuclear inflammatory cells. (c) Breast muscle with marked (grade 3) pathology characterised by widespread variation in myofibre size and shape and separation of myofibres by collagen. (d) Higher magnification showing degenerate (*) and necrotic (arrowhead) myofibres. Scale bar is 200 μm in (a) and (b) and 20 μm in (c) and (d).

**Figure 2.**
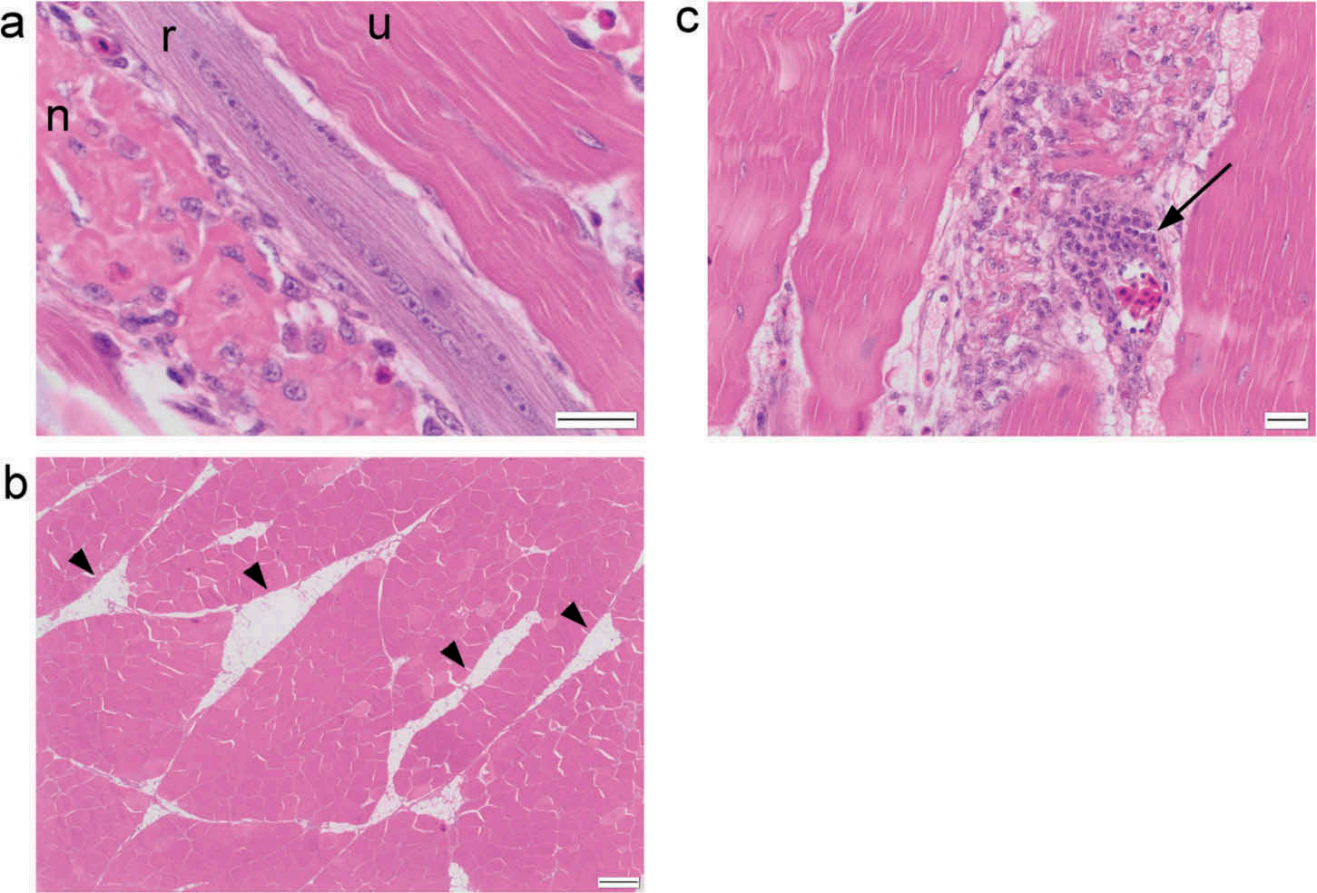
*(a) Longitudinal section of breast muscle showing an unaffected myofibre (u), a necrotic myofibre (n) and a regenerating myofibre (r). (b) Adipocyte “cords” (arrowheads) present within the breast muscle. (c) H&E stained section of breast muscle with prominent perivascular cuffing (arrow). Scale bar is 20 μm in (a) and (c) and 200 μm in (b)*.

The changes identified in the sections from both trials were comparable. They consisted of muscle degeneration, necrosis and regeneration spanning a spectrum of severity from mild to marked. The least severe changes consisted of occasional isolated myofibre degeneration (Figure 1(a)), characterised by myofibres exhibiting a fragmented or vacuolated sarcoplasm (“moth-eaten”), or a smooth, eosinophilic sarcoplasm with loss of cross-striations. Occasionally cells were shrunken with hyper-eosinophilic sarcoplasm. Angular myofibres were occasionally identified. Rarely, myofibres were invaded by mononuclear inflammatory cells (lymphocytes and macrophages) with fewer heterophils (Figure 1(b)). Rare myofibres had a basophilic streaking or stippling to the sarcoplasm. These mild changes were predominantly acute to subacute. At the other end of the spectrum some sections had very severely affected muscle tissue with few normal myofibres present (Figure 1(c)). In these sections, extensive myofibre degeneration and necrosis was present (Figure 1(d)), and there were numerous examples of regenerating myofibres characterised by multinucleate cells, nuclear rowing and sarcoplasm basophilia (Figure 2(a)). Increased numbers of eosinophilic strands of collagen were present in the perimysium and endomysium (fibrosis), sometimes separating individual myofibres. Some sections of muscle contained thick cords of adipose tissue separating myofibres and muscle fascicles (Figure 2(b)). Others contained clusters of mononuclear inflammatory cells which often surrounded blood vessels (cuffing) (Figure 2(c)). The changes identified can be summarised as mild to marked, multifocal to diffuse, polyphasic, acute to chronic myofibre degeneration and necrosis with regeneration, fibrosis, adipocyte infiltration and perivascular mononuclear cuffing.

There were no quantifiable differences in pathological changes between the muscles from the different treatments. Mean pathology scores for the conventional and low temperature treatments, respectively, were: severity, 1.78 and 1.80 (SED 0.221, *P = *0.996); presence of adipose tissue cords (back transformed), −1.17 (0.31) and −0.78 (0.46) (SED 0.763, *P = *0.634); and presence of perivascular mononuclear cell accumulation, −2.08 (0.13) and −0.83 (0.44) (SED 0.475, *P = *0.084). The numbers of sections with 25/75, 50/50 or 75/25% classified as acute/chronic were 6, 5, 5 for the conventional and 5, 8, 3 for the low treatments (*χ* ^2^ (2 df) 1.26, *P = *0.526).

## DISCUSSION

Commercial targets at 42 d for male broilers of this genotype are 3.0 kg (Aviagen, 2013) compared to 3.6 kg in this trial, differences that are probably associated with the low stocking density and small pens. These conditions enabled the broilers to achieve close to their potential growth rates on both treatments and may also have contributed to the lack of difference in performance between broilers on the two treatments as the comfort temperatures for the cold treatment are specified for commercial broilers stocked at a more conventional rate. Ambient temperatures on the two treatments diverged from 21 d onwards (Table 1) with a maximum difference of 7°C being attained by the end of the growing period. A potential source of changes in the biological effects of the thermal conditions imposed in the two experiments was differences in the forced convective regimes resulting from the chamber ventilation and air mixing (Mitchell, 1985*a*, 1985*b*). A marked forced convective environment in Experiment 1 induced behavioural responses associated with reduction in heat loss (e.g. huddling and fluffing of feather cover) whereas in Experiment 2, where air flow baffles reduced direct convective cooling, behaviours were consistent with a more homogeneous thermal environment. As there were no significant differences in body temperature responses in the two trials or in measures of performance or muscle quality then it is proposed that the forced convective environment did not influence the outcome other than in terms of bird behaviour and comfort.

Where birds are housed on litter under commercial conditions it is unlikely that the effective thermal load experienced by individuals would be as low as that experienced by birds in a pen experiment. Whereas rectal temperature was lower in the cool treatment, this may not reflect an actual “chilling” of the birds as the mean values were both in the normal expected range for broiler chickens of this age. Indeed the mean rectal temperature values for the conventional temperature group may be towards the higher end of the normal range and the cool treatment may have restored these to a more “normal value”. Mortality in both trials from 7 d was associated with ascites and was similar in both treatments. It is possible that the marked air movement in the climate chambers may have affected mortality from this cause in both trials in addition to the high growth rates of these birds.

The lack of differences in growth and feed intake between the temperature regimens was unexpected: it was assumed that broilers in the cool treatment would either grow more slowly or eat more feed. Four pens were therefore created in each climate chamber so that two pens could be managed to match the feed intake or growth of the alternative treatment if differences became apparent during the experiment. However, this was not the case and 3 birds from all 4 pens in each chamber were sampled.

Whereas muscle pathology was clearly observed in these birds, there were no detectable differences between the two temperature regimens implying that the low temperature treatment did not improve muscle characteristics. Plasma CK activity is a useful measure of muscle cell damage and was also similar in birds from both treatments. The hypothesis that muscle damage was caused by an inability to disperse metabolic heat in these fast-growing broiler chickens is therefore rejected. The alternative hypothesis that increasing levels of abnormalities in broiler breast muscle are related to genetic selection that does not address muscle cell function is consistent with evidence of substantial genetic variation for breast muscle quality (Le Bihan-Duval *et al*., 2008; Sandercock *et al*., 2009).

No differences in muscle quality were detected except that breast muscle from birds kept in the low temperature regimen was initially darker, more red and less yellow. The greater lightness and redness remained at 24 h, whereas yellowness was very variable and not significantly different. Comparable changes in breast muscle colour have occurred in short-term exposure of broilers to acute cold stress (Dadgar *et al*., 2012). However, to the authors’ knowledge, this is the first report of an association between long-term cold exposure and increased stiffness of breast muscle.

The histopathology identified in the chicken breast muscle was consistent with changes reported by other workers (Petracci *et al*., 2013*b*). Interestingly, in this study, the severity of the pathological changes identified in the muscle sections varied widely. In some sections, the myofibre degeneration and necrosis was marked and extensive, demonstrating the hallmarks of a “polyphasic” insult (the presence of acute and chronic changes) to muscle tissue, suggestive of a continuous or repeated insult. However, other sections contained only a small number of degenerative myofibres scattered throughout the tissue. Interestingly, the changes on each slide occasionally varied between sections of the same sample and in some cases, even within section. For example, very severe changes were present in one section but only mild or moderate changes in another section on the same slide, and sometimes one area of a section was affected but not another. This suggests that the myopathic changes are multifocal, rather than diffuse and that consistent sampling of multiple regions of the muscle from each bird is crucial.

The fact that there is considerable variation at the phenotypic level (Kuttappan *et al*., 2013*a*) suggests that genetic selection for the improvement of muscle quality traits would be effective. Conventional selection for low plasma CK activity in selection candidates or meat quality traits in sib tests could be conducted. Alternatively, DNA-based whole genome selection methods, where associations of meat quality traits and genome-wide single nucleotide polymorphisms are used to determine genomic breeding values for selection candidates (Meuwissen *et al*., 2001), could be the method of choice.

## Supplementary Material

Supplementary Table
