# Supplementary material for: Predicted optimum ambient temperatures for broiler chickens to dissipate metabolic heat do not affect performance or improve breast muscle quality
Source: Br Poult Sci. 2016 Feb 29;57(1):134–41. doi: 10.1080/00071668.2015.1124067 (PMC4784492; doi:10.1080/00071668.2015.1124067)
Supplement: Supplementary Table [file cbps_a_1124067_sm1083.docx]

**Supplementary Table**. *Feed intake and body weight from hatch to 42 d of age in male broiler chickens reared in a conventional or cool temperature regimen from 3 weeks of age*

| Trait | Conventional | Cool | SED | | Significance (*P*) | |  |
| --- | --- | --- | --- | --- | --- | --- | --- |
| *Mean body weight, g* | | | |  | |  | |
| 7 d | 182 | 182 | 4 | | 0.94 | |  |
| 14 d | 513 | 514 | 14 | | 0.95 | |  |
| 21 d | 1030 | 1047 | 11 | | 0.17 | |  |
| 28 d | 1799 | 1814 | 61 | | 0.82 | |  |
| 35 d | 2662 | 2589 | 42 | | 0.15 | |  |
| 42 d | 3608 | 3576 | 97 | | 0.75 | |  |
| *Feed intake, g/d/bird* | | | |  | |  | |
| 4-7 d | 27 | 26 | 0.7 | | 0.52 | |  |
| 8-14 d | 107 | 104 | 4.6 | | 0.50 | |  |
| 15-21 d | 103 | 104 | 1.0 | | 0.88 | |  |
| 21-28 d | 167 | 168 | 3.5 | | 0.77 | |  |
| 28-35 d | 196 | 200 | 5.8 | | 0.53 | |  |
| 35-42 d | 262 | 254 | 13.3 | | 0.64 | |  |
| *Mean feed conversion ratio (FCR), g feed/g body weight* | | | | | | | |
| 8-14 d | 0.89 | 0.81 | 0.066 | | 0.27 | |  |
| 15-21 d | 1.40 | 1.37 | 0.043 | | 0.44 | |  |
| 22-28 d | 1.52 | 1.56 | 0.066 | | 0.61 | |  |
| 29-35 d | 1.62 | 1.80 | 0.132 | | 0.20 | |  |
| 36-42 d | 1.71 | 1.76 | 0.141 | | 0.75 | |  |
